# Supplementary material for: Food security in rural South Africa: The role of household head demographics, crowding, and wealth
Source: PLOS Glob Public Health. 2026 Mar 25;6(3):e0006171. doi: 10.1371/journal.pgph.0006171 (PMC13016305; doi:10.1371/journal.pgph.0006171)
Supplement: S1 Table — (DOCX) [file pgph.0006171.s001.docx]

Food Security in Rural South Africa: The Role of Household Head Demographics, Crowding, and Wealth

Reneilwe G Mashaba^1^, Cairo B Ntimana^¶1,2^*, Katlego Mothapo^¶1^, Kurisani M Mabhedle^¶1^, Joseph Tlouyamma^¶1^ ,Kagiso P Seakamela^2^

^1^ DIMAMO Population Health Research Centre, University of Limpopo, Sovenga St, Polokwane 0727, South Africa

^2^ Department of Pathology, University of Limpopo, Sovenga St, Polokwane 0727, South Africa

^*^ Cairo B Ntimana

Email: cairo.ntimane@ul.ac.za

**S1 Table: Crude and adjusted odds ratios (OR) for factors associated with food insecurity in rural South African households**

| Variables | Crude OR (95% CI) | Crude P-value | Adjusted OR (95% CI) | Adjusted P-value |
| --- | --- | --- | --- | --- |
| Household Head Age |  |  |  |  |
| < 39 Years old | Ref | – | Ref | – |
| 40 – 69 Years old | 0.49 (0.39 – 0.61) | <0.001 | 0.43 (0.37 – 0.63) | <0.001 |
| ≥ 70 Years old | 0.30 (0.03 – 0.39) | <0.001 | 0.17 (0.10 – 0.31) | <0.001 |
| Household Head Gender |  |  |  |  |
| Male | Ref | – | Ref | – |
| Female | 1.13 (0.96 – 1.33) | 0.152 | 0.73 (0.52 – 1.04) | 0.082 |
| Household Head Marital Status |  |  |  |  |
| Single | Ref | – | Ref | – |
| Married | 1.34 (1.24 – 1.91) | <0.001 | 0.97 (0.34 – 0.87) | 0.890 |
| Divorced | 3.04 (1.77 – 4.23) | <0.001 | 5.39 (2.88 – 10.12) | <0.001 |
| Household Head Education |  |  |  |  |
| No formal education | Ref | – | Ref | – |
| Primary education | 1.18 (0.62 – 2.21) | 0.616 | 0.98 (0.51 – 1.87) | 0.957 |
| Secondary education | 1.19 (0.69 – 2.34) | 0.530 | 0.68 (0.38 – 1.24) | 0.210 |
| Tertiary education | 0.67 (0.19 – 2.34) | 0.532 | 0.40 (0.11 – 1.47) | 0.170 |
| Household Head Employment |  |  |  |  |
| EmployedSUPPLEMENTAL | Ref | – | Ref | – |
| Not employed | 1.02 (0.74 – 1.42) | 0.885 | 1.19 (0.84 – 1.70) | 0.332 |
| Household Crowding |  |  |  |  |
| Low crowding | Ref | – | Ref | – |
| Medium crowding | 1.39 (1.12 – 1.72) | 0.003 | 2.03 (1.37 – 3.02) | <0.001 |
| High crowding | 2.03 (1.65 – 2.49) | <0.001 | 2.62 (1.75 – 3.90) | <0.001 |
| Wealth Index Quintiles |  |  |  |  |
| Poor | Ref | – | Ref | – |
| Middle | 0.73 (0.59 – 0.90) | 0.003 | 0.73 (0.34 – 0.72) | 0.075 |
| Rich | 1.11 (0.92 – 1.34) | 0.269 | 0.50 (0.34 – 0.72) | <0.001 |
