# Supplementary material for: Food security in rural South Africa: The role of household head demographics, crowding, and wealth
Source: PLOS Glob Public Health. 2026 Mar 25;6(3):e0006171. doi: 10.1371/journal.pgph.0006171 (PMC13016305; doi:10.1371/journal.pgph.0006171)
Supplement: S1 Fig — (DOCX) [file pgph.0006171.s002.docx]

Food Security in Rural South Africa: The Role of Household Head Demographics, Crowding, and Wealth

Reneilwe G Mashaba^1^, Cairo B Ntimana^¶1,2^*, Katlego Mothapo^¶1^, Kurisani M Mabhedle^¶1^, Joseph Tlouyamma^¶1^ ,Kagiso P Seakamela^2^

^1^ DIMAMO Population Health Research Centre, University of Limpopo, Sovenga St, Polokwane 0727, South Africa

^2^ Department of Pathology, University of Limpopo, Sovenga St, Polokwane 0727, South Africa

^*^ Cairo B Ntimana

Email: cairo.ntimane@ul.ac.za

**
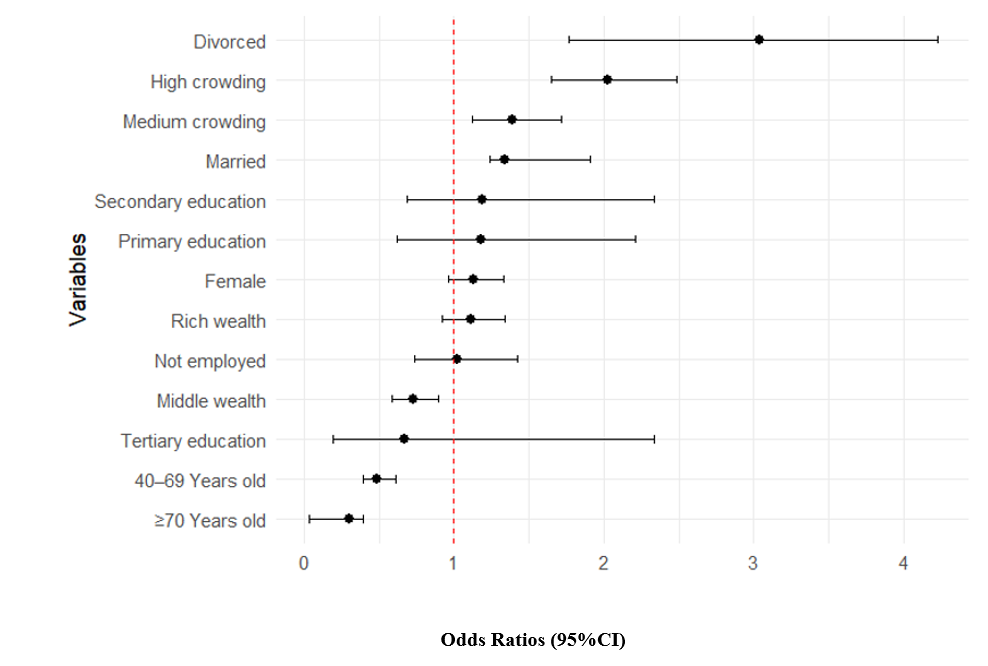
**

**S1 Fig:** Bivariate logistic regression of food insecurity status by household and household head characteristics
