# Supplementary material for: Food security in rural South Africa: The role of household head demographics, crowding, and wealth
Source: PLOS Glob Public Health. 2026 Mar 25;6(3):e0006171. doi: 10.1371/journal.pgph.0006171 (PMC13016305; doi:10.1371/journal.pgph.0006171)
Supplement: Attachment — Submitted filename: Comments_Plos_Food_insecurity.pdf [file pgph.0006171.s004.pdf]

<u>**COMMENTS**</u>

Article: Food Security in Rural South Africa: The Role of Household Head Demographics, Crowding, and Wealth

Thank you for the opportunity to review this paper.

Abstract

1. “This study aimed to investigate the association between household food security and the demographics of the household head, as well as household-level characteristics, in Limpopo Province, South Africa.” This sentence should be revised because, in its current form, it is unclear what the focal independent variables are. Additionally, it does not align well with the title.
2. “…used to describe the relationship between the binary dependent variable (Food secure = 0, Food insecure = 1) and the predictor variables.” What are the predictor variables?
3. Your cocnlusion is basically a repetition of the results.

Introduction

1. Food insecurity, defined as ‘insufficient accessibility or access to nutritious food or a limited/uncertain ability to buy food in socially acceptable ways is a persistent public health challenge [1,2].” Is your definition a direct quote? If so, note that the authors you are citing originally derived their definition from the American Dietetic Association. You should therefore reference it appropriately.
2. “In South Africa, the prevalence of food insecurity has been on the rise[6]. According to STATS SA, the proportion of households in South Africa that experienced moderate to severe food insecurity was estimated at 15,8% in 2019, 16,2% in 2022, and 19,7% in 2023 [7].” This reference seems to originate from a master’s thesis rather than the WHO as indicated, and it should be correctly cited.
3. “Furthermore, household and household head demographics have been reported to play a role in food security.” What does the literature say about this connection?

Methods

1. “The current study aimed to investigate the effect of the household head’s demographics on food security.” There needs to be consistency in this statement. How about wealth and crowding?
2. “To assess the relative socioeconomic status of households, a wealth index was constructed using the Principal Component Analysis (PCA).” How was this done? What were some of the variables used in deriving the variable?
3. Lines 73 – 74. I am curious why you chose to adapt 18 binary indicators from the HFIAS rather than using the original questions, given that the scale already includes 18 items. This approach is unclear and requires clarification. Additionally, is your scale valid and reliable?

4. Lines 81 – 83. Can you make reference to similar studies that use this criteria to measure crowding?
5. Please clarify how the binary food security variable was generated from the four original categories. The categories/classifications presented in Table 2 do not appear to align with those described in lines 77–79. It seems that you may have combined "marginal food security" with "high food security." What is the rationale or theoretical justification for this decision?
6. "Variables with high multicollinearity were removed from the regression model." Which variables?
7. Lines 96 – 100. I am curious is the authors used primary or secondary data. The detail information provided under this section suggests that the authors collected primary data.
8. Some typos exist in this se

Results

1. "Divorced household heads were associated with food insecurity (AOR=5.39; 95%CI:2.88-10.12; P<0.001)." What does AOR mean?
2. I recommend merging your tables. Do you really need table 2?

Discussion

1. The discussion lacks depth and primarily reiterates the results without a meaningful contextual analysis. What recoomendations do you have in terms of your findings?
2. The authors have not provided any limitations to the study.
3. Your conclusion once again is a repetition of your results. What are the implicatins of your findings?
4. There are numerous grammatical errors and typos throughout the paper, and it requires thorough proofreading to improve clarity and readability. For instance, "Household heard Gender" in tables 3 and 4.

References

1. There are some erros in the references. For example, reference 4 is not properly done. Please check all references.
