# Supplementary material for: Food security in rural South Africa: The role of household head demographics, crowding, and wealth
Source: PLOS Glob Public Health. 2026 Mar 25;6(3):e0006171. doi: 10.1371/journal.pgph.0006171 (PMC13016305; doi:10.1371/journal.pgph.0006171)
Supplement: Attachment — Submitted filename: point-by-point response.pdf [file pgph.0006171.s006.pdf]

Table of corrections

Reviewer 1

Location	Reviewer Comment	Change Made in Manuscript	Page and line number
Methods	The authors mentioned in the methodology that the study utilized secondary data collected from the Dikgale Mamabolo and Mothiba Health and Demographic Surveillance System (HDSS) now referred to as DIMAMOD HDSS. Also reiterated in line 66 "For this, 17,374 household heads were extracted from the DIMAMO PHRC database on 5th May 2025". If this is correct, I am wondering why the authors wrote the ethical considerations as though they obtained the ethical approval and obtained written consent from all 17,374 participants. The authors should kindly clarify this important aspect of the work to avoid confusion.	The study used secondary data, the reiteration on ethics, the authors we trying to outline how the data was collected. Now removed to improve clarity and avoid confusion.	
Results	The authors presented all their results in tables which is very informative, I opine that graphical presentation will enhance interpretation by providing a	Corrected as advised the authors have added graphical presentation to improve the clear and understanding of the data	

	clearer and more intuitive understating of the data.		
Introduction	Line 52. ……crowding and wealth index, in Limpopo province, South Africa (Preposition "in" omitted).	Added	Page 16, line 71
Discussion	Line 142. The preposition "in" is equally missing	Added	
	167: assist in elevating food insecurity [34–36]. (Should be alleviating).	Corrected as advised	
	Line 152-153: One study reported that as the educational attainment of the head of household increased, the level of household food security also improved [27]. Your result is not in tandem with this assertion, readers will be interested in possible reasons why? Authors should give robust explanation(s) to this and other results not in agreement with existing literature.	Corrected "The present study found that the household head's gender, educational status, and employment status did not significantly associate with food insecurity. Contrary to the findings of the present study, Kara and Kithu [36] reported that as the educational attainment of the head of household increased, the level of household food security also improved [36]. The discrepancies between the present study and that of Kara and Kithu [36] may be as a result of multiple confounding factors. Education measures years of schooling or the highest qualification	Page 14, line, 200-210

		and may not capture economic returns, such that household heads with similar educational levels may have different economic attainments [37]. As a result, some household heads, although not educated, may be informally employed, with purchasing power needed to improve food security. Lastly, the inconsistencies between the two studies may be due to different methodological approaches and localities [35]."	
Conclusion	Line194-195: The present study noted a low prevalence of food insecurity (3.51%) in the DIMAMO HDSS, Limpopo Province, South Africa, which is low when compared to national and regional estimates. (Recast to improve clarity)	Rephrased "The present study noted a low prevalence of food insecurity (3.51%). The low prevalence of food insecurity suggests that social protection mechanisms and local indigenous farming are a crucial barrier to households' food scarcity"	Page 13, line 275-277

Reviewer 2

Comment

I want to commend you on your expertly crafted manuscript, which adheres impeccably to the PLOS Global Public Health guidelines. The findings you have presented are not only highly relevant but also pivotal to advancing research in the crucial areas of food security and nutrition. This study powerfully underscores the urgent need for similar assessments in various regions around the globe. Even a small percentage of individuals grappling with food insecurity and poor nutrition can profoundly undermine the socio-economic development of families, communities, and indeed, entire nations. Your work sheds light on this vital issue and calls for action that could have far-reaching benefits

Remarks
Thank you

Reviewer 3

Location	Reviewer Comment	Change Made in Manuscript	Page and line number
Abstract	"This study aimed to investigate the association between household food security and the demographics of the household head, as well as household-level characteristics, in Limpopo Province, South Africa." This sentence should be revised because, in its current form, it is unclear what the focal independent variables are. Additionally, it does not align well with the title.	Rephrased "This study aimed to investigate how the demographics of the household head, household crowding, and wealth influence household food security in rural Limpopo Province, South Africa. "	Page 2, line 15 to 17.
	"…used to describe the relationship between the binary dependent variable (Food secure = 0, Food insecure = 1)	Included "The predictor variables (Household head's age, gender, marital status, education,	Page 2. Line 20-21.

	and the predictor variables." What are the predictor variables?	employment, household wealth index, and crowding)."	
	Your conclusion is basically a repetition of the results.	Rephrased "The present study noted a low prevalence of food insecurity (3.51%). The low prevalence of food insecurity suggests that social protection mechanisms and local indigenous farming are a crucial barrier to households' food scarcity. The age of the household head was reported to be a protective factor for food insecurity, with food insecurity decreasing as age increased. The protective role of older age highlights the importance of income stability provided by old-age grants; hence, similar financial safety mechanisms could be extended to unemployed younger household heads. Households in higher wealth quintiles were less likely to be food insecure. Household crowding was reported to be associated with food insecurity, where higher household crowding was associated with higher vulnerability. There's a need for family planning and livelihood interventions that aim to reduce economic challenges in crowded	Page 2 – 3. Line 29 – 41.

		households. Married and widowed household heads were also reported to be associated with food insecurity. There should be targeted interventions for widowed households, such as social support programs (food assistance and income-generating initiatives), to minimize food insecurity in this group with such a demographic. Future longitudinal studies should focus on the causal association between social protection, household composition, and food security to inform policy design at the disadvantaged local communities and provincial levels."	
Introduction	Food insecurity, defined as 'insufficient accessibility or access to nutritious food or a limited/uncertain ability to buy food in socially acceptable ways is a persistent public health challenge [1,2]." Is your definition a direct quote? If so, note that the authors you are citing originally derived their definition from the American Dietetic Association. You should therefore reference it appropriately.	Addressed as advised	Page 3. Line 47

	"In South Africa, the prevalence of food insecurity has been on the rise[6]. According to STATS SA, the proportion of households in South Africa that experienced moderate to severe food insecurity was estimated at 15,8% in 2019, 16,2% in 2022, and 19,7% in 2023 [7]." This reference seems to originate from a master's thesis rather than the WHO as indicated, and it should be correctly cited.	Addressed as advised	Page 3. Line 51 and 54.
	"Furthermore, household and household head demographics have been reported to play a role in food security." What does the literature say about this connection?	A systematic review conducted in Ethiopia reported that households headed by females, low educational attainment, and unemployment were associated with food insecurity, while an increase with age, higher education, and high wealth index were protective barriers for food insecurity [19]. Similarly, a study conducted in Nigeria noted similar findings with female-headed households being at risk of food insecurity [20].	Page 4. Line 65-68
Methods	"The current study aimed to investigate the effect of the household head's demographics on food security." There needs to be	The aim was corrected in the manuscript to cover all the aspects of wealth and crowding elsewhere in the manuscript but deleted in methods	Page 4. Line 84.

	consistency in this statement. How about wealth and crowding?		
	"To assess the relative socioeconomic status of households, a wealth index was constructed using the Principal Component Analysis (PCA)." How was this done? What were some of the variables used in deriving the variable?	To assess the relative socioeconomic status of households, a wealth index was constructed using principal component analysis (PCA). We included a set of household assets and amenities that are commonly used to measure long-term economic status. These included items such as cooking appliances, a refrigerator, a television, a bed, a table and chairs, bicycles, a car/bakkie, livestock, and access to electricity-dependent appliances. Only binary variables with variation were retained for the PCA.	Page 5. Line 88-92
	Lines 73 – 74. I am curious why you chose to adapt 18 binary indicators from the HFIAS rather than using the original questions, given that the scale already includes 18 items. This approach is unclear and requires clarification. Additionally, is your scale valid and reliable?	We have clarified the construction of the binary food security variable. "Food secure" and "mild food insecurity" were combined because both categories reflect worry or limited dietary quality, but do not involve reductions in food intake. In contrast, "moderate" and "severe" insecurity are characterized by compromised quantity and meal skipping. This distinction aligns with	-

	the conceptual structure of the HFIAS and previous studies that separate households with anxiety/quality issues from those with actual food deprivation. We have revised the Methods section to make this rationale clear.	
Lines 81 – 83. Can you make reference to similar studies that use this criteria to measure crowding?	Referenced	Page 5. Line 119.
Please clarify how the binary food security variable was generated from the four original categories. The categories/classifications presented in Table 2 do not appear to align with those described in lines 77–79. It seems that you may have combined "marginal food security" with "high food security." What is the rationale or theoretical justification for this decision?	For analysis, the four food security categories were converted to a binary variable (food secure = 0, food insecure = 1) by combining "food secure" with "mild food insecure". We adapted the food insecurity indicators from HFIAS because the dataset did not contain the original HFIAS questions. These items mapped closely to the frequency-based HFIAS domains, allowing us to construct a comparable measure. Although the adapted scale is not the full HFIAS, similar adaptations have been used in household surveys where only partial or modified items are available [23–25]	Page 5. Line 102-106

	"Variables with high multicollinearity were removed from the regression model." Which variables?	All variables had VIF values below the commonly used threshold of 10, indicating no problematic multicollinearity.	Page 6. Line 119-120
	Lines 96 – 100. I am curious is the authors used primary or secondary data. The detail information provided under this section suggests that the authors collected primary data.	The study used secondary data, the part that spoke about " A trained Research ….." the authors we trying to outline how the data was collected. Now removed to improve clarity.	Page 6, line 126-127
	Some typos exist in this se	Corrected throughout the document.	All manuscript
Results	"Divorced household heads were associated with food insecurity (AOR=5.39; 95%CI:2.88-10.12; P<0.001)." What does AOR mean?	Adjusted Odds Ratios	
	I recommend merging your tables. Do you really need table 2?	Table 2 removed.	
Discussion	The discussion lacks depth and primarily reiterates the results without a meaningful contextual analysis. What recommendations do you have in terms of your findings?	The discussion has been refined now outlining the implications of the study findings and recommendations form future studies	Page 13-16, line 189-263
limitations	The authors have not provided any limitations to the study.	The study was cross-sectional in nature the causal relationships between household/household head characteristics and food insecurity could not be established. In addition,	Page 16, line 265-273

		the relationships between household/household head characteristics and food insecurity are to be interpreted as correlations rather than causations. Based on the self-reported data collected through household surveys can result in a recall bias. The present study used secondary data. Hence, the study did not analyse for all possible determinants of food insecurity (i.e., dietary diversity, seasonal variations in food availability). The findings of the present study may not be generalized to other regions, since the study was conducted in the Capricorn region of Limpopo province. Additionally, the food insecurity measure was adapted from HFIAS and did not include all original questions, which may limit the scope of experiences captured. Nevertheless, the study provides valuable insights into the socio-demographic and structural factors influencing household food security in rural Limpopo.	

Conclusion	Your conclusion once again is a repetition of your results. What are the implicatins of your findings?	The present study noted a low prevalence of food insecurity (3.51%) in the DIMAMO HDSS, Limpopo Province, South Africa, which is low when compared to national and regional estimates. The low prevalence of food insecurity suggests that social protection mechanisms and local indigenous farming isare a crucial barrier forto households' food scarcity. The age of the household head was reported to be a protective factor for food insecurity, with food insecurity decreasing as age increased. The protective role of older age highlights the importance of income stability provided by old-age grants; hence, similar financial safety mechanisms could be extended to unemployed younger household heads. Households in higher wealth quintiles were less likely to be food insecure. Household crowding was reported to be associated with food insecurity, where higher household crowding was associated with higher vulnerability. TheresThere's a need for family planning and livelihood interventions that aim to reduce economic challenges in crowded households. Married and widowed	Page 16- 17. Line 277-290

		household heads were also reported to be associated with food insecurity. There was no association between gender, educational status, and employment status with food insecurity.There should be targeted interventions for widowed households, such as social support programs (food assistance and income-generating initiatives), to minimize food insecurity in this group with such a demographic. Future longitudinal studies should focus on the causal association between social protection, household composition, and food security to inform policy design at the disadvantaged local communities and provincial levels.	
	There are numerous grammatical errors and typos throughout the paper, and it requires thorough proofreading to improve clarity and readability. For instance, “Household heard Gender” in tables 3 and 4.	Corrected	
	There are some erros in the references. For example, reference 4 is not properly done. Please check all references.	Corrected	
