# Supplementary material for: Food security in rural South Africa: The role of household head demographics, crowding, and wealth
Source: PLOS Glob Public Health. 2026 Mar 25;6(3):e0006171. doi: 10.1371/journal.pgph.0006171 (PMC13016305; doi:10.1371/journal.pgph.0006171)
Supplement: Attachment — Submitted filename: point-by-point response_R3.docx [file pgph.0006171.s007.docx]

**Table of corrections**

**Reviewer 1**

| Location | Reviewer Comment | Change Made in  Manuscript | Page and line number |
| --- | --- | --- | --- |
| Abstract | “This study aimed to investigate the association between household food security and the demographics of the household head, as well as householdlevel characteristics, in Limpopo Province, South Africa.” This sentence should be revised because, in its current form, it is unclear what the focal independent variables are. Additionally, it does not align well with the title. | Rephrased “This study aimed to investigate how the demographics of the household head, household crowding, and wealth influence household food security in rural Limpopo Province, South Africa. “ | Page 2, line 15 to 17. |
|  | “…used to describe the relationship between the binary dependent variable (Food secure = 0, Food insecure = 1) | Included “The predictor variables (Household head’s age, gender, marital status, education, | Page 2. Line 20-21. |

|  | and the predictor variables.” What are the predictor variables? | employment, household wealth index, and crowding).” |  |
| --- | --- | --- | --- |
|  | Your conclusion is basically a repetition of the results. | Rephrased “The present study noted a low prevalence of food insecurity (3.51%). The low prevalence of food insecurity suggests that social protection mechanisms and local indigenous farming are a crucial barrier to households’ food scarcity. The age of the household head was reported to be a protective factor for food insecurity, with food insecurity decreasing as age increased. The protective role of older age highlights the importance of income stability provided by old-age grants; hence, similar financial safety mechanisms could be extended to unemployed younger household heads. Households in higher wealth quintiles were less likely to be food insecure. Household crowding was reported to be associated with food insecurity, where higher household crowding was associated with higher vulnerability. There's a need for family planning and livelihood interventions that aim to reduce economic challenges in crowded | Page 2 – 3. Line 29 – 41. |

|  |  | households. Married and widowed household heads were also reported to be associated with food insecurity. There should be targeted interventions for widowed households, such as social support programs (food assistance and income-generating initiatives), to minimize food insecurity in this group with such a demographic. Future longitudinal studies should focus on the causal association between social protection, household composition, and food security to inform policy design at the disadvantaged local communities and provincial levels.” |  |
| --- | --- | --- | --- |
| Introduction | Food insecurity, defined as  ‘insufficient accessibility or access to nutritious food or a limited/uncertain ability to buy food in socially acceptable ways is a persistent public health challenge [1,2].” Is your definition a direct quote? If so, note that the authors you are citing originally derived their definition from the American Dietetic Association. You should therefore reference it appropriately. | Addressed as advised | Page 3. Line 47 |

|  | “In South Africa, the prevalence of food insecurity has been on the rise[6]. According to STATS SA, the proportion of households in South Africa that experienced moderate to severe food insecurity was estimated at  15,8% in 2019, 16,2% in 2022, and 19,7% in 2023 [7].” This reference seems to originate from a master’s thesis rather than the WHO as indicated, and it should be correctly cited. | Addressed as advised | Page 3. Line 51 and 54. |
| --- | --- | --- | --- |
|  | “Furthermore, household and household head demographics have been reported to play a role in food security.” What does the literature say about this connection? | A systematic review conducted in Ethiopia reported that households headed by females, low educational attainment, and unemployment were associated with food insecurity, while an increase with age, higher education, and high wealth index were protective barriers for food insecurity [19]. Similarly, a study conducted in Nigeria noted similar findings with female-headed households being at risk of food insecurity [20]. | Page 4. Line 65-68 |
| Methods | “The current study aimed to investigate the effect of the household head’s demographics on food security.” There needs to be | The aim was corrected in the manuscript to cover all the aspects of wealth and crowding elsewhere in the manuscript but deleted in methods | Page 4. Line 84. |

|  | consistency in this statement. How about wealth and crowding? |  |  |
| --- | --- | --- | --- |
|  | “To assess the relative socioeconomic status of households, a wealth index was constructed using the Principal Component Analysis (PCA).” How was this done? What were some of the variables used in deriving the variable? | To assess the relative socioeconomic status of households, a wealth index was constructed using principal component analysis (PCA). We included a set of household assets and amenities that are commonly used to measure long-term economic status. These included items such as cooking appliances, a refrigerator, a television, a bed, a table and chairs, bicycles, a car/bakkie, livestock, and access to electricity-dependent appliances. Only binary variables with variation were retained for the PCA. | Page 5. Line 88-92 |
|  | Lines 73 – 74. I am curious why you chose to adapt 18 binary indicators from the HFIAS rather than using the original questions, given that the scale already includes 18 items. This approach is unclear and requires clarification. Additionally, is your scale valid and reliable? | We have clarified the construction of the binary food security variable. “Food secure” and “mild food insecurity” were combined because both categories reflect worry or limited dietary quality, but do not involve reductions in food intake. In contrast, “moderate” and “severe” insecurity are characterized by compromised quantity and meal skipping. This distinction aligns with | - |

|  |  | the conceptual structure of the HFIAS and previous studies that separate households with anxiety/quality issues from those with actual food deprivation. We have revised the Methods section to make this rationale clear. |  |
| --- | --- | --- | --- |
|  | Lines 81 – 83. Can you make reference to similar studies that use this criteria to measure crowding? | Referenced | Page 5. Line 119. |
|  | Please clarify how the binary food security variable was generated from the four original categories. The categories/classifications presented in Table 2 do not appear to align with those described in lines 77–79. It seems that you may have combined “marginal food security” with “high food security.” What is the rationale or theoretical justification for this decision? | For analysis, the four food security categories were converted to a binary variable (food secure = 0, food insecure = 1) by combining “food secure” with “mild food insecure”. We adapted the food insecurity indicators from HFIAS because the dataset did not contain the original HFIAS questions. These items mapped closely to the frequencybased HFIAS domains, allowing us to construct a comparable measure. Although the adapted scale is not the full HFIAS, similar adaptations have been used in household surveys where only partial or modified items are available [23–25] | Page 5. Line 102-106 |

|  | “Variables with high multicollinearity were removed from the regression model.” Which variables? | All variables had VIF values below the commonly used threshold of 10, indicating no problematic multicollinearity. | Page 6. Line 119-120 |
| --- | --- | --- | --- |
|  | Lines 96 – 100. I am curious is the authors used primary or secondary data. The detail information provided under this section suggests that the authors collected primary data. | The study used secondary data, the part that spoke about “ A trained Research …..” the authors we trying to outline how the data was collected.  Now removed to improve clarity. | Page 6, line 126-127 |
|  | Some typos exist in this se | Corrected throughout the document. | All manuscript |
| Results | “Divorced household heads were associated with food insecurity (AOR=5.39; 95%CI:2.88-10.12;  P<0.001).” What does AOR mean? | Adjusted Odds Ratios |  |
|  | I recommend merging your tables. Do you really need table 2? | Table 2 removed. |  |
| Discussion | The discussion lacks depth and primarily reiterates the results without a meaningful contextual analysis. What recommendations do you have in terms of your findings? | The discussion has been refined now outlining the implications of the study findings and recommendations form future studies | Page 13-16, line 189-263 |
| limitations | The authors have not provided any limitations to the study. | The study was cross-sectional in nature the causal relationships between household/household head characteristics and food insecurity could not be established. In addition, | Page 16, line 265-273 |

|  |  | the relationships between household/household head characteristics and food insecurity are to be interpreted as correlations rather than causations. Based on the self-reported data collected through household surveys can result in a recall bias. The present study used secondary data. Hence, the study did not analyse for all possible determinants of food insecurity (i.e., dietary diversity, seasonal variations in food availability). The findings of the present study may not be generalized to other regions, since the study was conducted in the Capricorn region of Limpopo province. Additionally, the food insecurity measure was adapted from HFIAS and did not include all original questions, which may limit the scope of experiences captured. Nevertheless, the study provides valuable insights into the sociodemographic and structural factors influencing household food security in rural Limpopo. |  |
| --- | --- | --- | --- |

| Conclusion | Your conclusion once again is a repetition of your results. What are the implicatins of your findings? | The present study noted a low prevalence of food insecurity  (3.51%) in the DIMAMO HDSS, Limpopo Province, South Africa, which is low when compared to national and regional estimates. The low prevalence of food insecurity suggests that social protection mechanisms and local indigenous farming isare a crucial barrier forto households’ food scarcity. The age of the household head was reported to be a protective factor for food insecurity, with food insecurity decreasing as age increased. The protective role of older age highlights the importance of income stability provided by old-age grants; hence, similar financial safety mechanisms could be extended to unemployed younger household heads. Households in higher wealth quintiles were less likely to be food insecure. Household crowding was reported to be associated with food insecurity, where higher household crowding was associated with higher vulnerability. TheresThere's a need for family planning and livelihood interventions that aim to reduce economic challenges in crowded households. Married and widowed | Page 16- 17. Line 277-290 |
| --- | --- | --- | --- |
|  |  | household heads were also reported to be associated with food insecurity. There was no association between gender, educational status, and employment status with food insecurity.There should be targeted interventions for widowed households, such as social support programs (food assistance and income-generating initiatives), to minimize food insecurity in this group with such a demographic. Future longitudinal studies should focus on the causal association between social protection, household composition, and food security to inform policy design at the disadvantaged local communities and provincial levels. |  |
|  | There are numerous grammatical errors and typos throughout the paper, and it requires thorough proofreading to improve clarity and readability. For instance, “Household heard Gender” in tables 3 and 4. | Corrected |  |
|  | There are some erros in the references. For example, reference 4 is not properly done. Please check all references. | Corrected |  |

**Reviewer 2/4.1**

| **Location** | **Reviewer Comment** | **Change Made in Manuscript** |
| --- | --- | --- |
| **Abstract** | The Conclusion section of the abstract seems the mere repetition of results and requires revision. The conclusion section should state key findings as per the specific objectives of study and doable recommendations to the point. | Thank you for your comment. The authors have revised the conclusion with doable recommendations |
|  | The terms bivariate and multivariate need to be replaced by bivariable and multivariable. | Corrected as advised |
|  | Data are plural (Data was versus Data are). | Corrected as advised |
|  | The sampling procedure and methods of data collection need to be stated. | Added “A cross-sectional study was conducted using routinely collected data from the DIMAMO Health and Demographic Surveillance System, including 17,374 household heads in rural Limpopo Province. Data were obtained through HDSS household surveillance using standardized questionnaires that included 12-month recall food security items and household socio-demographic characteristics” |
|  | All predictors need to be supported with measure of association (AOR) and 95% confidence interval consistently or described simply qualitatively. | We have added the (AOR) and 95% confidence interval |
|  |  |  |
| **Introduction** | Food insecurity definition should follow the standard definition of Food and Agriculture Organization (FAO) definition “food insecurity occurs when people lack regular access—because of financial, physical, or other resource constraints—to enough safe and nutritious food for normal growth, development, and an active healthy life | Rephrased “defined as suggested” |
|  | What is really not known about predictors of food insecurity across the globe? There is plenty of evidence on predictors of food insecurity. What gaps the present study wanted fill is not convincing? | Thank you for your comment. We have incorporated what is really not known about the predictors of food insecurity across the globe…In addition, we have rephrased the gap the study aims to fill. |
|  |  |  |
| **Methods** | The study setting should describe the agro-ecological characteristics including the pattern of rain fall as it relates to food production. | Added ad advised “The area lies within a semi-arid agro-ecological zone with warm summers, mild winters, and seasonal rainfall that falls mainly from October to March. Agricultural activities in this area depend on rainfall, and households rely largely on subsistence crop production and small-scale livestock farming. Consequently, rainfall variability directly influences local food production and household food availability “ |
|  | How feasible and valid is using a 12-months recall Food Security questions. | We clarified the rationale for using a 12-month recall food security measure in the Data preparation and instruments section, highlighting its suitability for capturing seasonal and chronic food insecurity in rural, rain-fed agricultural settings, while acknowledging potential recall bias |
|  | Is it scientifically sound to combine combining “food secure” with “mild food insecure”? | The decision to combine “food secure” and “mildly food insecure” categories follows established HFIAS and FIES guidance, which conceptualizes mild food insecurity as food-related anxiety rather than actual dietary deprivation. Prior studies and population-based surveys have applied similar binary classifications, particularly in low-prevalence settings, to improve analytical robustness and interpretability (Coates et al., 2007; Middlemass e al., 2021). |
|  | How assumptions of PCA were checked? | We have clarified how the assumptions of principal component analysis were assessed in the Wealth index construction section. Specifically, we examined variable variability and inter-item correlations, assessed sampling adequacy using the Kaiser–Meyer–Olkin measure, applied Bartlett’s test of sphericity, and selected components based on eigenvalues and scree plot inspection. These steps confirmed the suitability of the data for PCA-based wealth index construction. |
|  |  |  |
| **Results** | Title of a table and figure should be complete answering three basic questions (what, where, and when). | We have addressed this comment by revising table and figure titles to include the “what, where, and when” information where this was methodologically appropriate. For some analytical tables and figures (e.g., regression models), the study setting and time period are already clearly stated in the Methods section and repeated in table notes; therefore, restating all three elements in the title was not applicable |
|  | I suggest table 2 to be substituted with appropriate figure | Fig 1 and Fig 2 are the visualizations of Table S2 and S3 |
|  | I suggest tables 3 & 4 to be merged as crude analysis and adjusted analysis in one table using crude odds ratio and adjusted odds ratio respectively. | Thank you for your helpful suggestion. We agree that presenting the crude and adjusted odds ratios in a single table improves clarity and facilitates comparison. As suggested, we have merged the two tables into one consolidated table (now labeled **Table S1**), which includes both the crude and adjusted ORs, 95% CIs, and p-values for all variables. The revised table is included below and has been updated in the supplementary file. |
|  | • For crude analysis use “COR” instead of “OR”. |  |
|  | Line 134-135 “With regards to the wealth index, the household heads who were from the rich category were 0.50 times less likely to be associated with food insecurity (p<0.001) [AOR=0.50,95%CI: (0.34 – 0.72)] –the interpretation needs to be revised as follows. “The odds of food insecurity were reduced by 50% among study participants from rich wealth category compared with those form “poor” wealth category. | Thanks for the comment, revised as advised |
|  |  |  |
| **Discussion** |  |  |
|  | The first paragraph of the discussion section should first state the main aim of the study and paraphrase the key findings as per the specific objectives of the study before commencing the discussion of each key finding. | The first paragraph of the Discussion was revised to explicitly restate the study aim and summarise the key findings in line with the study objectives before discussing individual results. |
|  | Limitations of the study need to be discussed at the end of discussion in one paragraph | Corrected as advised |
|  | Implications of results need to be discussed as well. | We added a brief paragraph to the Discussion outlining the key policy and programmatic implications of the findings, focusing on social protection, household crowding, and targeted support for vulnerable household groups in rural setting |
| **Conclusion** | This section needs to summarize the key finding as per the specific aims of the study qualitatively with put repeating the actual figure, for instance, need of mentioning the prevalence of food insecurity as 3.51%. | Revised as advised |

**Reviewer 3**

**Comments**

On page 8, line 143, insert the figure number (currently it looks like Fig. Table S1.

**Correction**

Thanks for the comment, corrected as advised
