# Supplementary material for: Food security in rural South Africa: The role of household head demographics, crowding, and wealth
Source: PLOS Glob Public Health. 2026 Mar 25;6(3):e0006171. doi: 10.1371/journal.pgph.0006171 (PMC13016305; doi:10.1371/journal.pgph.0006171)
Supplement: Attachment — Submitted filename: Reviewer 5.docx [file pgph.0006171.s008.docx]

**Reviewer 5**

| Reviewer Comment | Response |
| --- | --- |
| Introduction:  - In lines 71-72 the author(s) stated that @For example, the role of household head demographics characteristics, household crowding, and wealth indices in influencing food security within rural South African communities remains not fully explored (This statement needs evidence and support; may be the authors can cite other studies or show what were the emphasis of previous food security studies in South Africa) | The authors have cited studies to support the evidence |
| Methods:  Line 87 – The authors indicated that they used the DIMAMO PHRC database. What is this database? How appropriate is it for the study? What are the methodologies of data collection in the DIMAMO PHRC database? | We thank the reviewer for this important comment. Additional information describing the DIMAMO Population Health Research Centre (PHRC) database, its purpose, and the methodology used for data collection has been added. The database is a longitudinal population-based surveillance system that collects demographic and socioeconomic data through regular household surveys conducted by trained fieldworkers. |
| The authors need to indicate what variables were extracted from the database, the timeline of the database, the rationale for extracting 17,374 household heads, and the population size in the database. | The Methods section has been revised to clearly indicate the variables extracted from the database, the study period covered, the total population under surveillance, and the rationale for selecting the analytic sample of 17,374 household heads. |
| Results  Line 144–145 – ‘There was a significant difference in food security by household wealth index… Why do the rich have higher food insecurity than the poor?’ | We appreciate the reviewer’s observation. A clarification has been added explaining that the wealth index reflects asset ownership rather than income or liquidity. In rural settings, households may own assets but still experience temporary financial constraints affecting food access. Multivariable analysis showed that higher wealth categories were associated with lower odds of food insecurity. |
| Line 150–151: Household head’s gender, educational status, and employment status were not associated with food insecurity. Is there any explanation for this? | We thank the reviewer for this observation. An explanatory statement has been added in the Discussion section to clarify that the lack of association may reflect contextual factors in rural South Africa. Specifically, social protection mechanisms such as government grants, subsistence farming, and informal livelihood activities may buffer households from food shortages irrespective of the household head’s gender, education level, or formal employment status. |
| Discussion: The fact that there is a low overall prevalence of food insecurity (3.51%) reduces the variation in the dependent variable. This will have effects in the model. How is this addressed in the paper? | We appreciate the reviewer’s important methodological comment. We have now addressed this in the Limitations and Strengths of the Study section. The manuscript now acknowledges that the relatively low prevalence of food insecurity (3.51%) may reduce variability in the dependent variable and potentially influence regression estimates. However, we clarify that the large sample size (n = 17,374 households) provided sufficient statistical power, and model diagnostics confirmed that the regression models remained stable. |
